# Effect of Caffeine and Flavonoids on the Binding of Tigecycline to Human Serum Albumin: A Spectroscopic Study and Molecular Docking

**DOI:** 10.3390/ph15030266

**Published:** 2022-02-22

**Authors:** Miroslav Sovrlić, Emina Mrkalić, Ratomir Jelić, Marina Ćendić Serafinović, Stefan Stojanović, Nevena Prodanović, Jovica Tomović

**Affiliations:** 1 Department of Pharmacy, Faculty of Medical Sciences, University of Kragujevac, Svetozara Markovića 69, 34000 Kragujevac, Serbia; msovrlic@medf.kg.ac.rs (M.S.); rjelic@uni.kg.ac.rs (R.J.); sstojanovic1987@gmail.com (S.S.); nensyprodanovic@yahoo.com (N.P.); jovicatomovic2011@gmail.com (J.T.); 2 Department of Science, Institute for Information Technologies, University of Kragujevac, Jovana Cvijića bb, 34000 Kragujevac, Serbia; 3 Department of Chemistry, Faculty of Science, University of Kragujevac, Radoja Domanovića 12, 34000 Kragujevac, Serbia; marina.cendic@pmf.kg.ac.rs

**Keywords:** spectroscopic measurements, docking simulations, tigecycline, human serum albumin, caffeine, flavonoids

## Abstract

Human serum albumin (HSA) has a very significant role in the transport of drugs, in their pharmacokinetic and pharmacodynamic properties, as well as the unbound concentration of drugs in circulating plasma. The aim of this study was to look into the competition between tigecycline (TGC) and alkaloid (ALK) (caffeine (CAF)), and flavonoids (FLAVs) (catechin (CAT), quercetin (QUE), and diosmin (DIO)) in binding to HSA in simulated physiological conditions using multiple spectroscopic measurements and docking simulations. Fluorescence analysis was used to find the binding and quenching properties of double HSA-TGC and triple HSA-TGC-CAF/FLAV systems. The conformational change of the HSA was analyzed using synchronous fluorescence spectroscopy, Fourier transform infrared spectroscopy, and circular dichroism. Obtained results of spectroscopic analyses indicate that triple complexes of HSA-TGC-CAF/FLAVs are formed without problems and have higher binding affinities than double HSA-TGC. In addition, TGC does not change the microenvironments around the tryptophan (Trp) and tyrosine (Tyr) residues in the presence of ALK and FLAVs. Ultimately, the binding affinity, competition, and interaction nature were explored by docking modeling. Computational outcomes are in good accordance with experimentally obtained results. Accordingly, concluding remarks may be very useful for potential interactions between common food components and drugs.

## 1. Introduction

**Figure 1:**
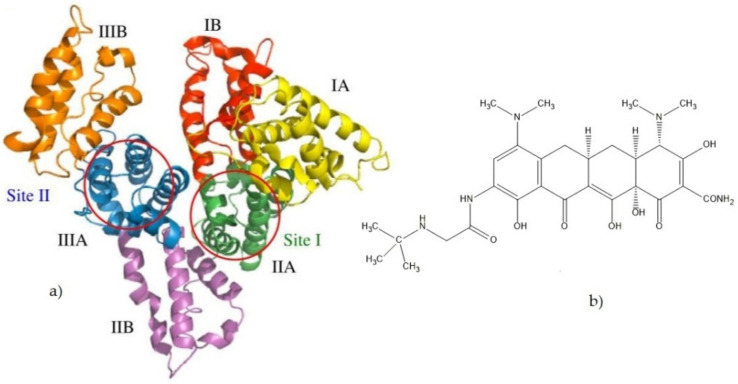
(**a**) Crystal structure of HSA. The subdivision of HSA into domains (I-III) and subdomains (**a**,**b**) is indicated, and the approximate locations of site I and site II are shown. Atomic coordinates were taken from the PDB entry 1UOR. The illustration was performed with PyMOL [4] (**b**). Chemical structure of tigecycline.

Human serum albumin (HSA), the most abundant protein in plasma, is constituted by a single chain of 585 residues arranged in a globular heart-shaped conformation (Figure 1) containing three homologous domains usually indicated as I, II, and III. Each domain includes two separate subdomains (A and B) [1]. HSA has the capability of binding reversibly to a large variety of drugs via its binding sites. Crystal structure analysis indicates that hydrophobic cavities in subdomains IIA and IIIA in albumin are the principal regions of ligand binding sites for aromatic and heterocyclic drugs [2,3]. It is of great importance from the viewpoint of pharmaceutical sciences to clarify the structure, function, and properties of HSA–drug complexes.

In clinical practice, the simultaneous administration of two or more drugs in the treatment of diseases is becoming a more common phenomenon. Therefore, clinically relevant drug-drug interactions at the level of binding to HSA are quite frequent. Antibiotics, such as tigecycline, are very often used to treat bacterial infections. A large part of the general population daily consumes drinks containing caffeine and meals containing these flavonoids. Consequently, the risk of interactions at the level of binding to HSA is much higher. In that way, one drug may alter the pharmacological or toxicological effect of another drug. So, there is a great clinical interest in investigating the effects of caffeine, flavonoids, and many other competitors on the binding of TGC to HSA [5,6] in the future.

Tigecycline (Figure 1) is the first of a new class of antimicrobials, the glycylcycline, containing the central four-ring carbocyclic skeleton, with substitution of an N-alkyl-glycylamido group at the D-9 position [7]. It possesses pharmacodynamic and pharmacokinetic properties different from other tetracyclines. In our previous paper, all the binding parameters of interaction between tigecycline and HSA were calculated [8]. However, investigations on the interaction of tigecycline with plasma protein and the effects of alkaloids and dietary flavonoids on tigecycline binding to plasma protein have not been reported so far. Alkaloid and flavonoid compounds, a class of natural drugs with high biological activity, are abundant in plants in nature. These compounds show many biological and therapeutical properties, such as antioxidant, antiviral, anticancer, anti-inflammatory, and heart disease protective activities. Numerous studies have been conducted regarding these compounds [9,10,11,12,13,14,15,16,17,18,19,20].

Caffeine, a purine alkaloid, is the most frequently consumed psychoactive substance due to the belief that it stimulates the central nervous system (CNS) [9]. When consumed in sufficient doses, caffeine is believed to influence the body’s water excretion or to enhance reactions to some psychostimulants like nicotine [10], even in moderate doses. Caffeine, as the main component of coffee, may also cause an increase in homocysteine concentration in serum, which may increase the risk of cardiovascular disease [11]. Catechins, known as flavan-3-ols, with strong antioxidant properties, have a wide range of applications in the pharmaceutical, agricultural, and food industries [12]. Catechins also showed prooxidative, cytotoxic, and phytotoxic activities, as negative effects [13,14].

Quercetin is one of the most abundant flavonoids in the human diet. It is an extensively studied molecule due to its complex mechanism of action and numerous pharmacological effects on the human organism [15]. Besides its antioxidant properties, it also displays anti-carcinogenic, anti-inflammatory, cardioprotective, neuroprotective, and anti-atherogenic activities [16]. Diosmin, (3′,5,7-trihydroxy-4′-methoxyflavone-7-rhamnoglucoside) is the active component of many drugs, especially ones used in the treatment of various blood vessel disorders. It has also recently been investigated for potential applications in cancer therapeutics [17,18].

**Figure 2:**
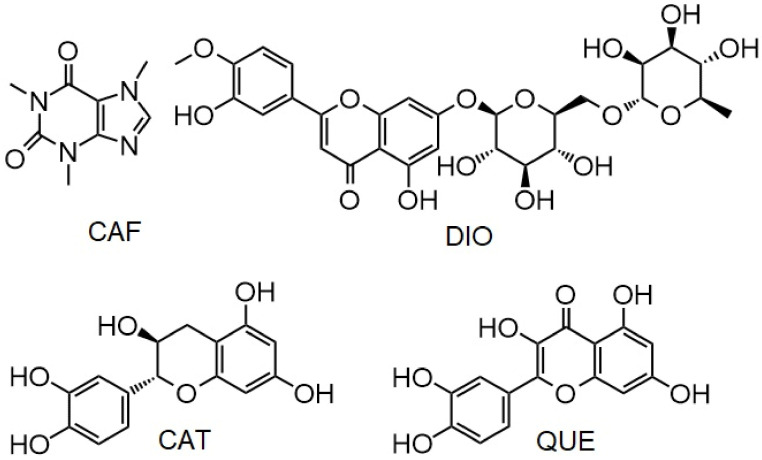
Chemical structures of examined ALK and FLAVs.

This study investigates the effects of the ALK (caffeine (CAF)) and FLAVs (catechin (CAT), quercetin (QUE) and diosmin (DIO)) (Figure 2) on binding tigecycline (TGC) to HSA, as well as their effects on the structure of the active site and the nature of interactions, in order to find out how food that is rich in these compounds can affect the use of TGC.

## 2. Results and Discussion

### 2.1. UV–Vis Absorption Spectra

UV–Vis absorption spectroscopy is a convenient and effective method for the steady-state study of protein-drug interactions and complex formation [21]. All observed changes (hypochromic/hyperchromic effect and/or red/blue shift) in UV-Vis spectra during titration may provide evidence of the existing interaction mode between drugs and HSA [22,23,24].

**Figure 3:**
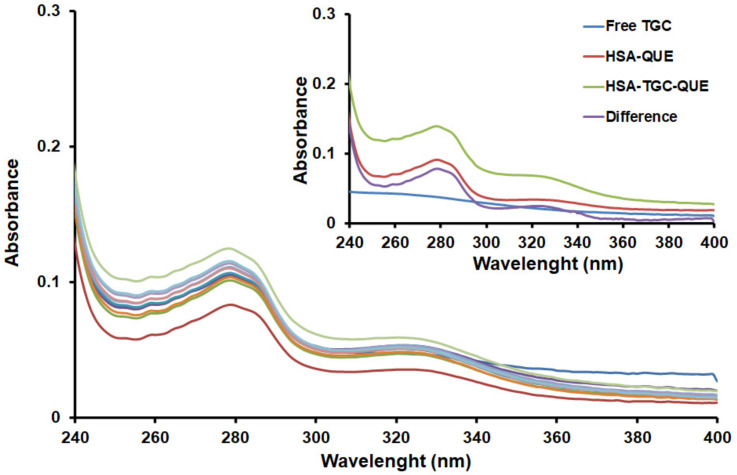
UV–Vis absorption spectra of HSA-QUE (1:1) in the absence and presence of increasing amounts of TGC (T = 298 K, pH = 7.4). [HSA] = [QUE] = 2 µM; [TGC] = 0, 1 *×* 10^−^^6^, 2 × 10^−^^6^, 3 × 10^−^^6^, 4 × 10^−^^6^, 5 × 10^−^^6^, 6 × 10^−^^6^, 7 × 10^−^^6^, 8 × 10^−^^6^, 9 × 10^−^^6^, 1 × 10^−^^5^ M. Inset: absorption spectrum of HSA-QUE; absorption spectrum of TGC only; absorption spectrum of HSA-TGC-QUE (1:2:1) complex; difference between absorption spectrum of HSA-TGC-QUE complex and free TGC.

In this work, we observed the change in the UV–Vis absorption spectra of the HSA-TGC-CAF/FLAV (QUE, CAT and DIO) system in the presence of varying concentrations of TGC. The contribution of TGC and competing for drugs (CAF and FLAVs (QUE, CAT and DIO)) was subtracted from the results in all complexes. Three aromatic amino acid residues (Trp, Tyr, and Phe) are responsible for the weak absorption peak around 280 nm (Figure 3 and Supplementary Materials Figures S1–S3). The absorption peak around 280 nm is raised, caused mainly by the transition p–p* of aromatic amino acid residues in HSA. As the concentration of TGC increases, the absorption intensity of HSA-CAF/FLAV (QUE, CAT and DIO) decreases, while the concentration of CAF/FLAVs (QUE, CAT and DIO) remains the same, suggesting the interactions between HSA-CAF/FLAV (QUE, CAT and DIO) and TGC through a static quenching mechanism. Furthermore, the UV difference spectra of HSA-TGC-CAF/FLAV (QUE, CAT and DIO) and absorption spectra of TGC have lower values than the HSA-CAF/FLAV (QUE, CAT and DIO) system. The dynamic quenching only affects the excited states of the fluorophores and, thus, no changes in the absorption spectra are expected. However, the ground state complex formation will result in perturbation of the absorption spectrum of the fluorophore [25]. We also found here a similar phenomenon observed with the fluorescence method. Based on these UV–Vis spectral results, it is evident that the progressive decrease in absorbance together with the increase in the concentration of TGC confirms the formation of triple protein–drug-drug complexes between HSA, TGC and CAF/FLAVs (QUE, CAT and DIO).

### 2.2. Fluorescence Quenching Measurements

**Figure 4:**
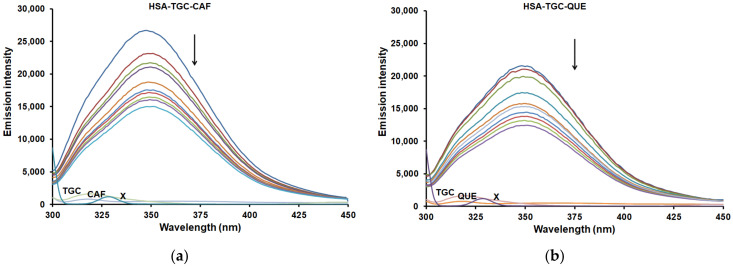
Fluorescence emission spectra of HSA-TGC (T = 298 K, pH = 7.4) in the presence of (**a**) CAF and (**b**) QUE. [HSA] = 2 µM, [CAF] = 2 µM, [QUE] = 2 µM and [TGC] = 0, 1 × 10^−6^, 2 × 10^−6^, 3 × 10^−6^, 4 × 10^−^^6^, 5 × 10^−^^6^, 6 × 10^−6^, 7 × 10^−6^, 8 × 10^−6^, 9 × 10^−6^, 1 × 10^−^^5^ M. X represents buffer only.

Most of the drugs bind with high affinity to one of the two sites, I and II, located in subdomains IIA and IIIA, respectively [2,3]. The presence of the Trp and Tyr amino acids in the structure of HSA causes its intrinsic fluorescence. The emission spectrum of HSA is primarily detected by excitation at 295 nm wavelength from the single Trp residue in subdomain IIA [25,26]. One of the main characteristics of the intrinsic fluorescence of HSA is that it is very sensitive to its microenvironment. Decreases in the intrinsic fluorescence (quenching) of HSA can occur even if there is a little change in the local surroundings of the HSA molecule, such as biomolecular binding, protein conformation, and denaturation. Therefore, the change in the intrinsic fluorescence intensity of HSA arises from variation in the environment around the Trp residue when small molecules are bound to HSA [27]. In this work, the effect of CAF and FLAVs (QUE, CAT and DIO) on the binding of TGC to HSA was investigated. In our previous work, it was concluded that Sudlow’s site I, located in subdomain IIA of HSA, was the primary binding site of TGC [24]. It was published that the primary binding site of CAF and FLAVs (QUE, CAT and DIO) was located in the same binding site [28,29,30,31], indicating that CAF and FLAVs share the same binding sites in the HSA with TGC. Figure 4 and Supplementary Materials Figures S4 and S5 show the fluorescence quenching of HSA-CAF/FLAV (QUE, CAT and DIO) in the presence of varying concentrations of TGC. At the excitation wavelength of 295 nm, it can be seen that HSA has a strong fluorescence emission peak at around 350 nm. As the concentration of TGC increased, the internal emission fluorescence of the HSA-CAF/FLAV (QUE, CAT and DIO) system gradually decreased, indicating the formation of the HSA-TGC-CAF/FLAV (QUE, CAT and DIO) complex. Furthermore, there were no significant changes in the maximum emission wavelength and shape of the peaks, implying that the binding of TGC to the HSA-CAF/FLAV (QUE, CAT and DIO) system caused no significant change in the microenvironment on the Trp residues in the HSA IIA subdomain.

### 2.3. Quenching Mechanism

Fluorescence quenching mechanism may be classified as dynamic, static, or combined quenching (both static and dynamic). Dynamic quenching implies collisional encounters between the fluorophore and quencher, and static quenching implies the formation of a nonfluorescent complex between the quencher and fluorophore. The fluorescence intensity was corrected for absorption of exciting light and re-absorption of emitted light according to Equation (1) [25]

(1) $$F_{cor}=F_{obs}\;\times \;10^{\frac{A_{ex}+A_{em}}{2}}$$

where *F_cor_* and *F_obs_* are the fluorescence intensity corrected and observed, respectively, and *A_ex_* and *A_em_* are the absorbance of the system at the excitation and emission wavelengths, respectively. The Stern–Volmer equation, Equation (2), [25] was used to analyze the quenching mechanism of HSA in the presence of CAF or FLAVs in ternary HSA-TGC-ALK/FLAV systems.

(2) $$\frac{F_{0}}{F}=1+K_{q}\tau_{0}\left[Q\right]=1+K_{\mathrm{SV}}\left[Q\right]$$

where *F_0_* is the emission intensities in the absence of the quencher, F is the emission intensities in the presence of the compound, *K*_SV_ is the Stern–Volmer quenching constant, *k*_q_ is the bimolecular quenching rate constant, *τ*_0_ is the average lifetime of the biomolecule without quencher where (*τ*_0_ = 10^−8^ s) and [Q] is the concentration of the quencher. Supplementary Materials Figure S6 shows the Stern–Volmer plots for the HSA-ALK/FLAVs fluorescence quenching by increasing concentrations of TGC. A linear Stern–Volmer plot indicates that only one mechanism of quenching occurs, static or dynamic [32,33]. The values of *K*_SV_ obtained from the slopes of the fit lines and *k*_q_ for the interaction of TGC with the HSA-ALK/FLAVs system are shown in Table 1. It can be observed that values of *K*_SV_ for the ternary HSA-TGC-ALK/FLAVs system were higher than that of the binary HSA-TGC system implying that the presence of ALK/FLAVs enhanced the interaction between HSA and TGC. However, the Stern–Volmer plot is not sufficient to define per se the quenching type, and therefore we analyzed the value of *k*_q_. The values of *k*_q_ were also greater than 2.0 × 10^10^ M^−^^1^s^−1^, which is the maximum scatter collision quenching constant of various quenchers with the biopolymers. These results indicate that the probable quenching mechanisms of the intrinsic fluorescence of HSA were not initiated by dynamic collision, but by the formation of ground state complexes. It is evident here that the quenching mechanism is a static quenching process originating from the formation of double and triple systems [33].

### 2.4. Binding Constant and Number of Binding Sites

**Figure 5:**
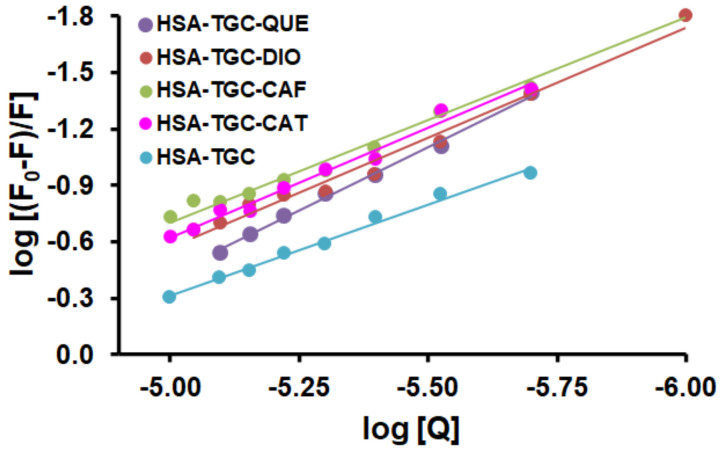
Logarithmic plots of the fluorescence quenching of HSA by TGC in the presence of CAF/FLAVs (QUE, CAT and DIO) at 298 K.

The binding affinity of the drug to HSA is a very important factor for the pharmacokinetic and pharmacodynamic properties of the drug [34,35]. Accordingly, the value of the binding constant (*K*_a_) is significant in understanding the distribution of a drug in plasma. In addition, the cellular uptake is proportional to the unbound concentration of drugs in circulating plasma. The decreased values of *K*_a_ cause weak binding and enhance the concentrations of free drug in the plasma leading to a short lifetime or poor distribution, while the increased values of *K*_a_ indicate a strong binding which can decrease the concentrations of free drug in plasma and then reduce the pharmacologic effects [34]. Figure 5 displays the plots of log (*F_0_* − *F*)/*F* against log[Q] for the HSA-CAF/FLAVs (QUE, CAT and DIO) system in the absence and presence of increasing concentrations of TGC. The binding constants (*K*_a_) and the number of binding sites (*n*) can be calculated by the double logarithm equation:

(3) $$\log\frac{F_{0}-F}{F}=\log K_{a}+n\log\left[Q\right]$$

and are listed in Table 1. The degree of binding of the drug to albumin may influence the rate of clearance of metabolites and their delivery to receptors in cells and tissues [36]. Based on the conventional concept, the cellular uptake is proportional to the unbound fraction of drugs [37]. According to this hypothesis, the distribution of the drug is proportional to the free concentration of unbound drug in circulating plasma. The decrease in the binding constant in the presence of competing drugs would shorten the storage time of the drug in plasma and a larger fraction of free drug available to act on target tissues would be present in plasma. If a drug is highly bound to serum albumins, the fraction of free drug may be reduced, and hence the effect of the drug may consequently be decreased [38]. As shown in Table 1, the presence of CAF, CAT, QUE, and DIO increased the binding constant of the HSA-TGC system. We found that the presence of competing drugs increased the affinity of TGC for HSA. Therefore, TGC could be stored and transported better by HSA in the presence of competing drugs. This also implies that CAF/FLAVs (QUE, CAT and DIO) can reduce the free concentration of unbound TGC and its pharmacological effect. Obtained values of n indicate the existence of only one binding site for TGC towards HSA in the absence or presence of CAF/FLAVs (QUE, CAT and DIO). There is an option that the two drugs can bind independently to different sites in the HSA molecule and there are no changes in the binding affinity of the drug to HSA (independent binding). As already reported, the primary binding site of CAF and FLAVs (QUE, CAT and DIO) is located in site I (subdomain IIA) [28,29,30,31] as well as TGC [24]. Therefore, competitive interference is expected between TGC and CAF/FLAVs (QUE, CAT and DIO) accompanied by a decrease in binding affinity of TGC to HSA in the presence of competing for drug (CAF/FLAVs (QUE, CAT and DIO)). If there were competitive binding (displacement of alkaloids or flavonoids by drug binding) then the binding constant of the drug to albumin would be significantly reduced compared to the constant when only drug and albumin are present in the solution. This does not happen in our case, see Table 1. In contrast, the Ka values obtained for triple complexes (HSA-TGC-CAF/FLAV (QUE, CAT, and DIO)) are higher than those obtained for double complexes (HSA-TGC), indicating that the binding affinity of TGC to HSA increased in the presence of CAF/FLAVs (QUE, CAT and DIO) (non-competitive interference). This simultaneous binding of two drugs increased the accessibility of the existing binding sites and hence increased the binding affinity of TGC to HSA. This may be caused by their simultaneous bond in different regions in subdomain IIA, which is large enough to accommodate multiple ligands at the same time [35]. As well, it may be explained as the result of conformational changes within the ligand, changes in the size and shape of the subdomain IIA in the presence of CAF/FLAVs (QUE, CAT and DIO). In addition, Huang et al. claim that the presence of fatty acids in subdomain IIA enhances the binding affinity of drugs to HSA by inducing a conformational change of HSA and creating a new subsite [39]. The results showed that TGC could be stored and transported better by HSA in the presence of CAF/FLAVs (QUE, CAT and DIO). This may lead to a decrease in the free concentration of unbound TGC in plasma. The binding stoichiometry between HSA and TGC was roughly 1:1 despite the presence of the competing drugs, except in the case of QUE leading to an increase in the value of n to 1.35. The claim that drugs with a higher binding constant can displace the drug with a lower binding constant is not valid in these experiments. There is no direct correlation between the degree of displacement and the binding constants of the two simultaneously used drugs here [35]. This effect probably depends on the nature of the drug (TGC) and the competing drugs (CAF and FLAVs (QUE, CAT and DIO)). Two drugs administered simultaneously can often have interactions at the level of binding to HSA, and some of these interactions may have clinical significance. Therefore, there is a great interest in analyzing the effect of CAF, QUE, DIO, and CAT and other competing drugs on the binding of TGC to HSA.

### 2.5. Synchronous Fluorescence Spectra

**Figure 6:**
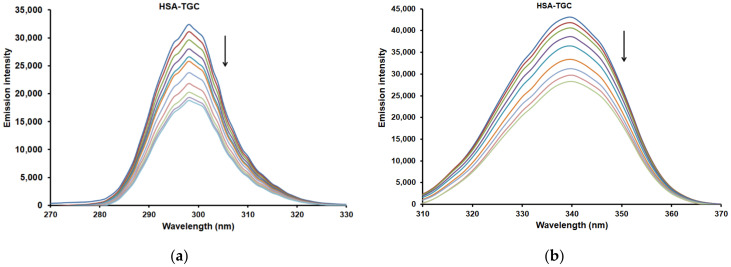
The effect of TGC on the synchronous fluorescence emission spectra of HSA (T = 298 K, pH = 7.4): (**a**) Δλ = 15 nm and (**b**) Δλ = 60 nm. [HSA] = 2 µM and [TGC] = 0, 1 × 10^−^^6^, 2 × 10^−^^6^, 3 × 10^−^^6^, 4 × 10^−^^6^, 5 × 10^−^^6^, 6 × 10^−^^6^, 7 × 10^−^^6^, 8 × 10^−^^6^, 9 × 10^−^^6^, 1 × 10^−^^5^ M.

Synchronous fluorescence is a very useful tool to explore the polarity change around the chromophore microenvironment by measuring the possible shift of the maximum emission wavelength. Synchronous fluorescence of HSA can provide characteristic information around tyrosine (Tyr) and tryptophan (Trp) residues when the scanning wavelength intervals (Δλ) are fixed at 15 nm and 60 nm, respectively [40]. The red or blue shift in the maximum fluorescence emission (wavelength) of HSA indicates enhanced hydrophilicity or hydrophobicity of the microenvironment around Tyr or Trp residues, respectively [41]. Figure 6 shows the synchronous fluorescence spectra of the binary HSA-TGC system. It is obvious that with increasing concentrations of TGC, the fluorescence intensity decreases regularly. These results confirm that the fluorescence quenching occurred. Further, there is no shift in the maximum emission wavelength at both 15 and 60 nm, which implies that the binding of TGC does not affect the conformation of the chromophore region [42].

**Figure 7:**
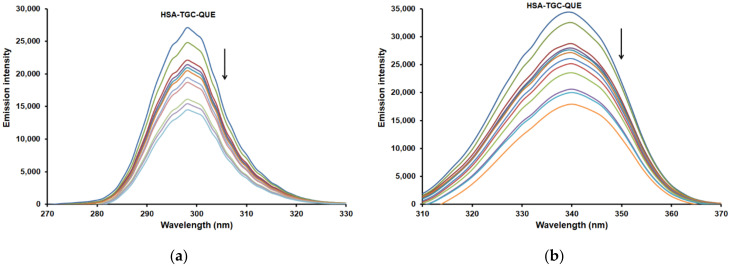
The effect of QUE on the synchronous fluorescence emission spectra of HSA-TGC system (T = 298 K, pH = 7.4): (**a**) Δλ = 15 nm and (**b**) Δλ = 60 nm. [HSA] = 2 µM, [QUE] = 2 µM and [TGC] = 0, 1 × 10^−6^, 2 × 10^−6^, 3 × 10^−6^, 4 × 10^−6^, 5 × 10^−6^, 6 × 10^−6^, 7 × 10^−6^, 8 × 10^−6^, 9 × 10^−6^, 1 × 10^−5^ M.

Also, similar results were obtained for the ternary HSA-TGC-CAF/FLAV (QUE, CAT and DIO) systems (see Figure 7 and Supplementary Materials Figures S7–S9). The emission maximum of the Tyr and Trp residues showed no significant shift, and accordingly, the interaction of HSA-TGC with CAF/FLAVs (QUE, CAT and DIO) showed no obvious effect on the conformation of the micro-region of Tyr and Trp [42].

### 2.6. Circular Dichroism Measurements

**Figure 8:**
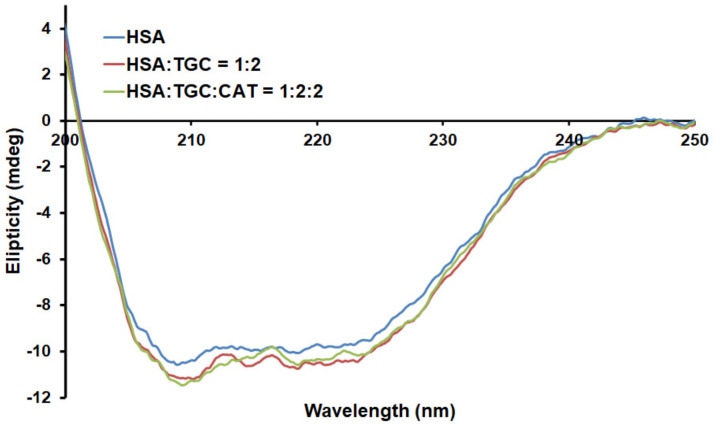
CD spectra of HSA with or without TGC and CAT (T = 298 K, pH = 7.4), respectively. The concentrations of HSA are 2 µM, and the molar ratios of TGC and CAT to HSA are 2:1 and 2:2:1.

The circular dichroism technique was used to show possible conformational changes that occurred due to protein-ligand interaction. CD spectra for HSA in the absence and presence of TGC for binary and TGC and CAT in ternary systems are shown in Figure 8. Two negative minima were observed at 208 and 222 nm and represent α- helix structure transition of π → π^⁎^ and n → π*. Both CD spectra (for the binary and ternary systems) were very similar in signal and shape, indicating that the binding of TGC in binary or TGC and CAT in ternary systems had a negligible effect on the secondary structure of the protein [43], which does not exclude a change in protein microenvironment.

### 2.7. Fourier Transform Infrared Spectroscopy (FT-IR)

The investigation of the secondary structure of HSA was performed using the FT-IR spectroscopic technique. The protein amide I band at 1650–1654 cm^−1^ and amide II band at 1548–1560 cm^−1^ are attributed to the secondary structure of all proteins [44]. Supplementary Materials Figure S10 shows FT-IR spectra of HSA both in the absence and the presence of TGC and CAT. No changes were observed in free HSA compared with HSA-TGC or HSA-TGC-CAT systems, suggesting no conformational changes in the HSA protein.

### 2.8. Docking Analysis

As already mentioned, it was published that TGC, CAF and FLAVs (QUE, CAT and DIO) shared common binding sites (I and II, located in subdomains IIA and IIIA) in the HSA protein [28,29,30,31]. Having performed individual docking (details described in [8] with a flexible Trp214 (this amino acid was used as it directly influences HSA-TGC-CAF/FLAV (QUE, CAT and DIO) bonding)) we performed sequential docking to check those claims [45,46]. In the usual individual docking procedure, we docked a single ligand with the receptor. However, with the help of sequential docking, it is possible to dock more than one ligand simultaneously [45]. This helps in detecting binding sites. Firstly, favored binding sites of triple HSA-TGC-CAF and HSA-TGC-FLAVs (QUE, CAT and DIO) regarding the double HSA-TGC system were examined. Secondly, competition, binding affinities, and binding constants of TGC with CAF and FLAVs (QUE, CAT and DIO) with HSA were investigated on the established favored side. Thirdly, the nature of interactions was analyzed. All tested triple HSA-TGC-CAF and HSA-TGC-FLAV (QUE, CAT and DIO) models have a significant increase in binding energy value (ΔG) regarding double system HSA-TGC (Table 2). It can be observed that ΔG values for site I (HSA-TGC = −24.36 kJ·mol^−1^, HSA-TGC-CAF = −24.89 kJ·mol^−1^, HSA-TGC-CAT = −25.60 kJ·mol-1, HSA-TGC-QUE = −30.75 kJ·mol^−1^ and HSA-TGC-DIO = −26.40 kJ·mol^−1^) are much higher than for site II (HSA-TGC = −14.60 kJ·mol^−1^, HSA-TGC-CAF = −15.35 kJ·mol^−1^, HSA-TGC-CAT = −16.65 kJ·mol^−1^, HSA-TGC-QUE = −18.78 kJ·mol^−1^ and HSA-TGC-DIO = −17.36 kJ·mol^−1^).

**Figure 9:**
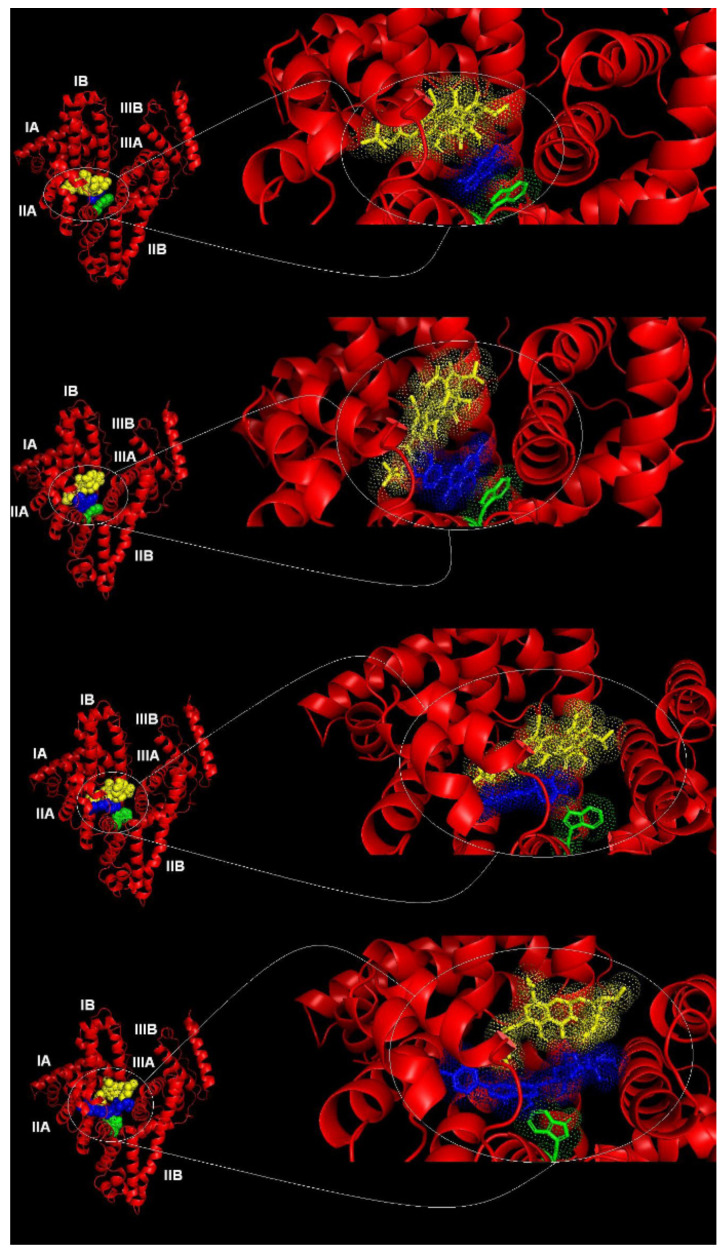
First = HSA-TGC-CAF; second = HSA-TGC-QUE; third = HSA-TGC-CAT; fourth = HSA-TGC-DIO (CAF and FLAVs (QUE, CAT and DIO) colored blue, TGC colored yellow and TRP214 colored green).

In comparison to the double HSA-TGC system (ΔG = −24.36 kJ·mol^−1^, K_i_ = 5.44 × 10^−5^ mol·dm^−3^ [8]) all triple HSA-TGC-CAF and HSA-TGC-FLAV (QUE, CAT and DIO) models have higher binding affinity and lower inhibition constants (Table 2). The lowest one is in the case of HSA-TGC-CAF (ΔG = −24.89 kJ·mol^−1^ with corresponding inhibition constant K_i_ = 4.33 × 10^−5^ mol·dm^−3^). It is followed by HSA-TGC-CAT (ΔG = −25.60 kJ·mol^−1^ with corresponding inhibition constant K_i_ = 3.27 × 10^−5^ mol·dm^−3^) and HSA-TGC-DIO (ΔG = -26.40 kJ·mol^−1^ with corresponding inhibition constant K_i_ = 2.37 × 10^−5^ mol·dm^−3)^ systems. The HSA-TGC-QUE has the highest binding energy (ΔG = −30.75 kJ·mol^−1^ with corresponding inhibition constant K_i_ = 4.13 × 10^−6^ mol·dm^−3^). Consequently, docking calculations are consistent with experimental results and predict tighter binding affinities of the triple HSA-TGC-FLAVs model in comparison to double HSA-TGC (Table 2). According to computer modeling, triple systems: HSA-TGC-CAF, HSA-TGC-QUE, HSA-TGC-CAT and HSA-TGC-DIO are located in site I (subdomain IIA) in an environment close to Trp214 residue. In Figure 9, sequential docking shows competitive possibility between TGC and CAF/FLAVs (QUE, CAT and DIO). The obtained computational energies of triple HSA-TGC-CAF and HSA-TGC-FLAVs (QUE, CAT and DIO) are higher than in the double HSA-TGC model, which indicates that the binding affinity of TGC to HSA increased in the presence of CAF/FLAVs (QUE, CAT and DIO). Sequential docking predicts the possible expansion of the existing binding site, so it can be assumed that subdomain IIA is large enough to accommodate multiple ligands at the same time.

**Figure 10:**
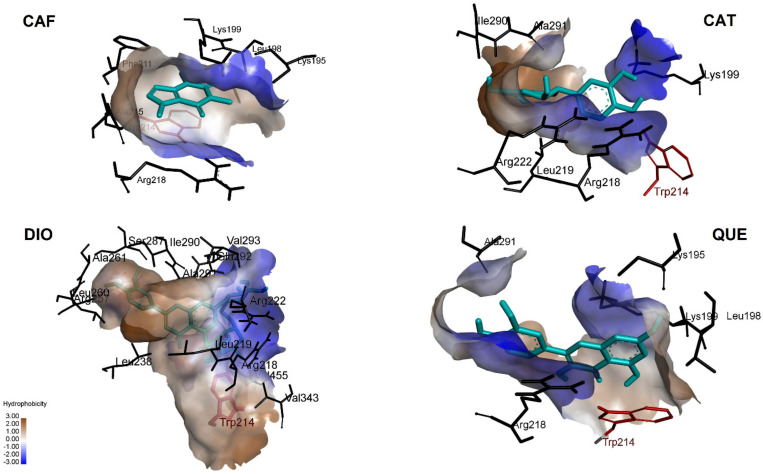
Hydrophobic contributions in HSA-TGC-ALK and HSA-TGC-FLAV.

**Figure 11:**
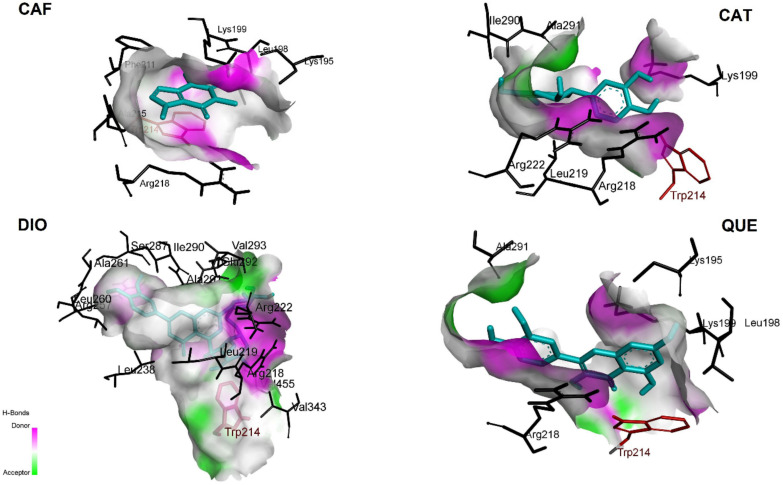
Contribution of H-bonds in HSA-TGC-ALK and HSA-TGC-FLAV.

Since sequential docking of triple HSA-TGC-CAF and HSA-TGC-FLAVs (QUE, CAT and DIO) shows the better possibility of inhibition and higher binding affinities, we studied these systems additionally in the sense of their interactive nature. Figure 10 and Figure 11 show hydrophobic and H-bond contributions. Generally, the CAF/FLAVs (QUE, CAT and DIO) core within the HSA was buried in the binding site using conventional H-bonds and π-cation electrostatics. CAF in HSA-TGC-CAF is surrounded primarily by the residues Lys195, Leu 198, Lys199, Phe211, Trp214, Ala215, Arg218. CAF is strongly positioned to interact with Trp214 through π-sigma, with Lys195, Leu198, Ala215, Arg218 through π-alkyl and with Lys 199 via π-cation interactions. The primary binding site was a hydrophobic cavity (Figure 10) formed by Lys195, Leu198, Lys199, Arg218 and Trp214 in subdomain IIA.

In the HSA-TGC-CAT model, the CAT area of bonding to HSA includes conventional hydrogen bonds (Figure 10) by Lys 199 (donor), Trp214, Arg218 (donor), Arg222 (donor), Ile290 (acceptor), and Ala291 (acceptor). The contribution of π-sigma comes from Leu 260 residue. Docking results of HSA-TGC-QUE show that QUE binding area contains Trp214 (π-π-stacked), Arg218 (π-sigma), Lys199 (π-cation), Lys195 (conventional hydrogen bond) and Ala291 (conventional hydrogen bond). HSA-TGC-DIO encompasses DIO π-alkyl connections from Trp214, Leu 219, Leu238, Leu260, Ala261, Val455 and π-sigma connection from Ile290. Further, DIO interacts with Arg218, Arg222, Glu292, Val293, Arg257 and Ser287 through conventional hydrogen bonds (Figure 11).

## 3. Materials and Methods

### 3.1. Materials

Fatty-acid-free human serum albumin (HSA, A1887), tigecycline hydrate (TGC, PZ0021), phosphate-buffered saline (PBS, P4417), catechin hydrate (CAT, 22110), and quercetin (QUE, Q4915) were purchased from Sigma–Aldrich Chemical Company (St. Louis, MO, USA) and used without further purification. Caffeine (CAF, 326356) was purchased from the Carlo Erba reagents. Diosmin (DIO, D3525) was purchased from Sigma Aldrich (Fluka). All starting materials were high-purity grade and used as purchased without further purification. The deionized water was used for preparing all solutions.

### 3.2. Solutions

HSA (2 × 10^−5^ M) was dissolved in PBS solution (0.01 M PBS, pH 7.4, 0.0027 M KCI, 0.137 M NaCl). The stock solution of TGC (3.413 × 10^−4^ M) was prepared by dissolving them in ethanol and then diluted to 8.538 × 10^−5^ M with a solution of PBS. The stock solutions of CAF and FLAVs (1 × 10^−3^ M) were dissolved in ethanol and diluted with a solution of PBS. The DIO (1 × 10^−3^ M) was dissolved in a small amount of DMSO (concentration of DMSO in HSA solution was at 0.1–1.5% (*v*/*v*)) and diluted with the solution of PBS.

### 3.3. Apparatus

#### 3.3.1. Ultra-Violet Spectroscopy

The UV/Vis absorption measurements were performed on a Perkin Elmer UV/Vis Lambda 365 spectrophotometer equipped with a 1 cm path length cell. All spectra were measured at room temperature in wavelengths ranging from 200 to 500 nm. The concentration of HSA was fixed at 2 μM, and the concentrations of covaried from 0 to 1 × 10^−5^ M. In the competitive experiments concentrations of HSA and CAF or FLAVs (QUE, CAT and DIO) were set at 2 μM, while the TGC was gradually added (from 0 to 1 × 10^−5^ M).

#### 3.3.2. Fluorescence Spectroscopy

2. D fluorescence spectra were collected on an RF-1501 PC spectrofluorometer (Shimadzu, Japan) with excitation at 295 nm, using a 150 W Xenon lamp source, 1.0 cm quartz cells and a thermostatic bath. Fluorescence spectra were recorded at 298 K in the range of 300–450 nm. The widths of the excitation and emission slit widths were both fixed at 10 nm. Synchronous fluorescence spectra were collected on an RF-6000 spectrofluorometer (Shimadzu, Japan) at 298 K. The wavelength interval (Δλ) for the synchronous scan spectra varied between 15 and 60 nm to assess the alterations in the microenvironment surrounding the aromatic amino acid residues Tyr and Trp, respectively. Double system. Synchronous fluorescence spectra of the HSA-TGC system were measured at 298 K. In all experiments, the concentration of the HSA was kept at 2 μM, while the concentration of the TGC varied from 0 to 1 × 10^−5^ M.

Triple systems. Experimental details of the interaction of HSA-TGC-CAF and HSA-TGC-FLAVs systems (FLAVs = CAT, DIO, QUE) were observed at 298 K. The UV–vis and fluorescence spectra of HSA (2 μM) and HSA-CAF and HSA-FLAVs (molar ratio 1:1) were recorded in the absence and presence of increasing amounts of TGC (0 to 1 × 10^−5^ M). The resultant mixtures were incubated at 298 K.

#### 3.3.3. Circular Dichroism Studies

The examination of conformational changes was performed through recording CD spectra on a JASCO J-815 spectropolarimeter (JASCO, Japan) at the room temperature of 298 K. For the far UV CD spectra (200–260 nm) the HSA concentration was 2 μM and TGC bound to HSA in the absence and presence of catechin (each 4 μM) were investigated for binary and ternary systems.

#### 3.3.4. Fourier Transform Infrared Spectroscopy (FT-IR)

The FT-IR spectra were recorded on a Perkin Elmer FT-IR spectrometer with a spectral resolution of 2 cm^−1^ and 24 scans. The spectra of free HSA and HSA in binary and ternary systems were compared and evaluated for any conformational changes. The concentrations of HSA and TGC/CAT were 2 μM and 4 μM, respectively.

#### 3.3.5. Molecular Docking

Tigecycline (TGC), ALK: caffeine (CAF) and FLAVs: catechin (CAT), quercetin (QUE) and diosmin (DIO) were downloaded from the PubChem Compound database (https://pubchem.ncbi.nlm.nih.gov/, accessed on 21 December 2020). Available protein 3D structure of HSA (PDB code 1HK1) was acquired from the Protein Data Bank (PDB) (RCSB PDB: Homepage). Docking processes were carried out using Autodock 4.2 [47,48]. Details of Autodock 4.2 individual docking procedure together with protein and ligands preparation so as to set up a flexible part, considered Trp214, are described elsewhere [8]. After the analysis of individual docking, sequential docking was performed [45,46]. In the procedure of sequential docking, the first ligand was docked, and the complex was saved as a single file, where the first ligand was considered as a part of the receptor. Docking was then carried out on this complex with the second ligand. The analysis of the 1HK1-TGC-CAF/1HK1-TGC-FLAVs (QUE, CAT and DIO) complex models was based on the hydrogen bonds and hydrophobic interaction. The values of ΔG binding and inhibition constants (Ki) were obtained from ADT after inspecting the results of the docking calculations. For the visualization of the docking results, a free version of the Discovery Studio Visualizer 3.5.0 Accelrys Software Inc. [49] and PyMOL [50] was used.

## 4. Conclusions

The research presented in this paper reveals that the effect of examined CAF and FLAVs (QUE, CAT and DIO) has a positive influence on binding TGC to HSA in simulated physiological conditions by multiple spectroscopic methods and docking simulations. In more detail, it was shown through the fluorescence quenching measurements that CAF and FLAVs (QUE, CAT and DIO) share IIA subdomain in HSA with TGC drug. There is also no significant change in the microenvironment on the Trp residues in the IIA subdomain as well as in hydrophilicity or hydrophobicity around it. Furthermore, triple complexes of HSA-TGC-CAF/HSA-TGC-FLAVs (QUE, CAT and DIO) are formed without problems and have higher binding affinities than double HSA-TGC. Docking experiments with HSA protein were performed, indicating a good correlation with experimental results. In comparison to the standard TGC, computational work also confirmed that examined ALK (CAF) and FLAVs (QUE, CAT and DIO) bind preferentially to sub-domain IIA of HSA and affinities of triple systems are higher than double ones (higher free energies and lower inhibition constants). Sequential docking predicts that subdomain IIA is large enough to accommodate multiple ligands at the same time. Once again, hydrogen bonds and hydrophobic interactions are responsible for the relatively strong binding between tested compounds and the HSA receptor. We believe that this cognition can lead to a better understanding of the ALK/FLAVs pharmaceutical potential and the influence of other drugs on the protein binding processes. This could certainly serve as a foundation for some future examinations.

## Figures and Tables

**Table 1:** The interaction parameters of the binary (HSA-TGC) and ternary (HSA-TGC-CAF/FLAVs (QUE, CAT and DIO), HSA: CAF/FLAVs (QUE, CAT and DIO) = 1:1) systems.

System [a]	*K*_SV_ × 10^−4^ [b]	*k*_q_ × 10^−12^ [c]	R^2^ [d]	*K*_a_ × 10^−5^ [b]	n	R^2^
HSA-TGC	5.00 ± 0.03	5.00	0.996	0.18 ± 0.03	0.9	0.991
HSA-TGC-CAF	1.87 ± 0.04	1.87	0.977	0.573 ± 0.06	1.09	0.985
HSA-TGC-CAT	2.57 ± 0.06	2.57	0.989	1.71 ± 0.07	1.17	0.983
HSA-TGC-QUE	3.96 ± 0.05	3.96	0.987	22.30 ± 0.02	1.35	0.996
HSA-TGC-DIO	2.47 ± 0.05	2.47	0.986	1.98 ± 0.08	1.17	0.982

[a] 298 K; [b] M^−1^; [c] M^−1^s^−1^; [d] R is the correlation coefficient.

**Table 2:** ΔG[a] values of site I (subdomain IIA) versus site II (subdomain IIIA). The inhibition constants of site I (subdomain IIA).

System [b]	Autodock		Ki[c]	Ref.
	Site I	Site II		
HSA-TGC	−24.36	−14.60	5.44 × 10^−5^	[8]
HSA-TGC-CAF	−24.89	−15.35	4.33 × 10^−5^	This work
HSA-TGC-CAT	−25.60	−16.65	3.27 × 10^−5^	
HSA-TGC-QUE	−30.75	−18.78	4.13 × 10^−6^	
HSA-TGC-DIO	−26.40	−17.36	2.37 × 10^−5^	

[a] kJ·mol−1; [b] 298 K; [c] mol·dm−3; Accordingly, the site I (subdomain IIA) of HSA was chosen as it binds TGC-CAF and TGC-FLAVs (QUE, CAT and DIO) tighter than site II (subdomain IIIA). Therefore, from that point only subdomain IIA was taken into consideration. Table 2 shows a more favored energy site with appropriate inhibition constants.

## Data Availability

Data is contained within the article and Supplementary Materials.

## Supplementary Materials

The following supporting information can be downloaded at: https://www.mdpi.com/article/10.3390/ph15030266/s1, Figures S1–S3 UV–Vis spectra of HSA-TGC in the presence of ALK and FLAVs, Figure S4. Fluorescence emission spectra of HSA-TGC-CAT system, Figure S5. Fluorescence emission spectra of HSA-TGC-DIO system, Figure S6. Stern-Volmer plots of the fluorescence quenching of HSA-TGC system by ALK and FLAVs, Figure S7. Synchronous fluorescence emission spectra of HSA-TGC-CAF system, Figure S8. Synchronous fluorescence emission spectra of HSA-TGC-CAT system, Figure S9. Synchronous fluorescence emission spectra of HSA-TGC-DIO system, Figure S10. FT–IR spectra of HSA in presence and absence of TGC and CAT.
