# Supplementary material for: Effect of Caffeine and Flavonoids on the Binding of Tigecycline to Human Serum Albumin: A Spectroscopic Study and Molecular Docking
Source: Pharmaceuticals (Basel). 2022 Feb 22;15(3):266. doi: 10.3390/ph15030266 (PMC8951011; doi:10.3390/ph15030266)
Supplement: Supplementary file 1 [file pharmaceuticals-15-00266-s001.zip › pharmaceuticals-1556828-supplementary.pdf]

Effect of caffeine and flavonoids on the binding of tigecycline to HSA: A spectroscopic study and molecular docking

Miroslav Sovrlić [1], Emina Mrkalić [2,*], Ratomir Jelić [1], Marina Ćendić Serafinović [3], Stefan Stojanović [1], Nevena Prodanović [1], Jovica Tomović [1]

[1] Department of Pharmacy, Faculty of Medical Sciences, University of Kragujevac, Svetozara Markovića 69, Kragujevac 34000, Serbia; msovrlic@medf.kg.ac.rs
[2] Department of Science, Institute for Information Technologies, University of Kragujevac, Jovana Cvijića bb, Kragujevac 34000, Serbia; emina.mrkalic@pmf.kg.ac.rs
[3] Department of Chemistry, Faculty of Science, University of Kragujevac, Radoja Domanovića 12, Kragujevac 34000, Serbia; marina.cendic@pmf.kg.ac.rs

* Correspondence: emina.mrkalic@pmf.kg.ac.rs (E.M)

Supplementary material

Content

1.1. **Figure S1-3** UV–Vis spectra of HSA-TGC in the presence of **ALK** and **FLAVs** 2

1.2. **Figure S4.** Fluorescence emission spectra of **HSA-TGC-CAT** system 3

1.3. **Figure S5.** Fluorescence emission spectra of **HSA-TGC-DIO** system 4

1.4. **Figure S6.** Stern-Volmer plots of the fluorescence quenching of **HSA-TGC** system by **ALK** and **FLAVs** 4

1.5. **Figure S7.** Synchronous fluorescence emission spectra of **HSA-TGC-CAF** system 5

1.6. **Figure S8.** Synchronous fluorescence emission spectra of **HSA-TGC-CAT** system 5

1.7. **Figure S9.** Synchronous fluorescence emission spectra of **HSA-TGC-DIO** system 6

1.8. **Figure S10.** FT–IR spectra of **HSA** in presence and absence of **TGC** and **CAT** 6

1.1. UV–Vis spectra of HSA-TGC in the presence of ALK and FLAVs

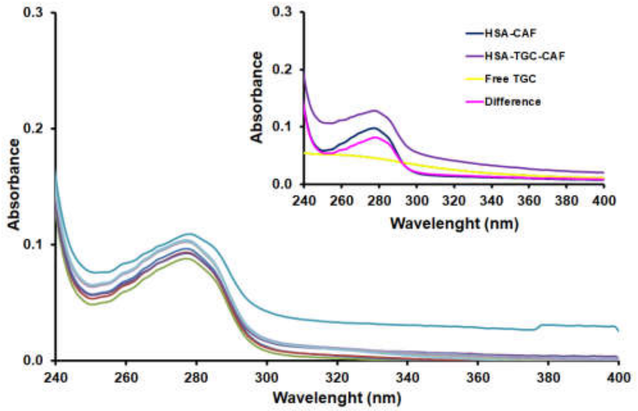

Figure S1. UV–Vis absorption spectra of HSA-CAF (1:1) in the absence and presence of increasing amounts of TGC (T = 298 K, pH = 7.4). [HSA] = 2 μM; [CAF] = 2 μM; [TGC] = 0 to 1 x 10^{-5} M. Inset: absorption spectrum of HSA-CAF; absorption spectrum of TGC only; absorption spectrum of HSA-TGC-CAF (1:2:1) complex; difference between absorption spectrum of HSA-TGC-CAF complex and free TGC.

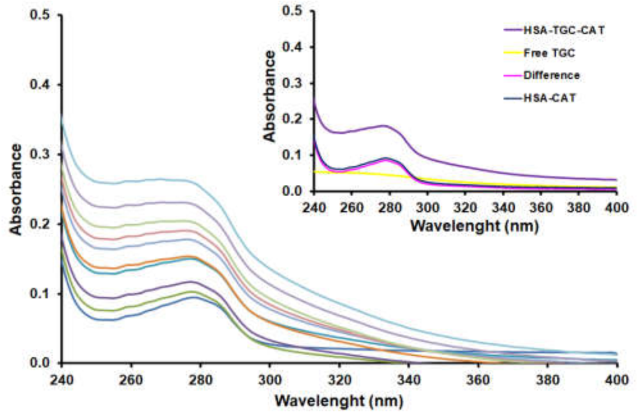

Figure S2. UV–Vis absorption spectra of HSA-CAT (1:1) in the absence and presence of increasing amounts of TGC (T = 298 K, pH = 7.4). [HSA] = 2 μM; [CAT] = 2 μM; [TGC] = 0 to 1 x 10^{-5} M. Inset: absorption spectrum of HSA-CAT; absorption spectrum of TGC only; absorption spectrum of HSA-TGC-CAT (1:2:1) complex; difference between absorption spectrum of HSA-TGC-CAT complex and free TGC.

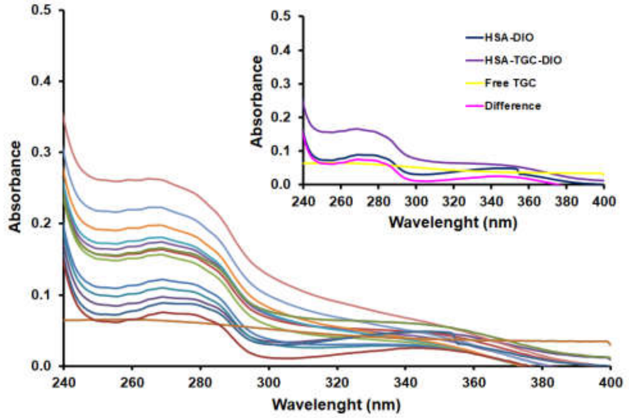

Figure S3. UV–Vis absorption spectra of HSA-DIO (1:1) in the absence and presence of increasing amounts of TGC (T = 298 K, pH = 7.4). [HSA] = 2 μM; [DIO] = 2 μM; [TGC] = 0 to 1 x 10^{-5} M. Inset: absorption spectrum of HSA-DIO; absorption spectrum of TGC only; absorption spectrum of HSA-TGC-DIO (1:2:1) complex; difference between absorption spectrum of HSA-TGC-DIO complex and free TGC.

1.2. Fluorescence emission spectra of HSA-TGC-CAT system

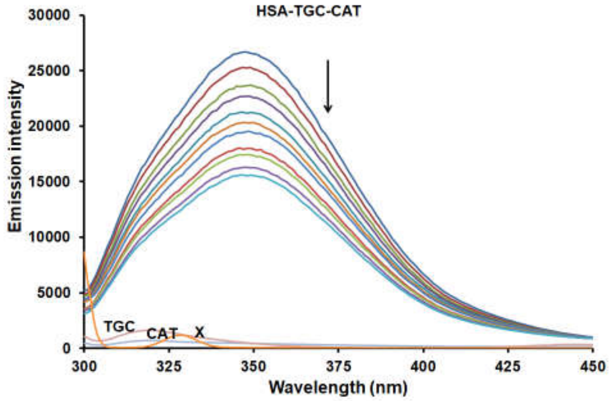

Figure S4. Fluorescence emission spectra of HSA-TGC (T = 298 K, pH = 7.4) in the presence of CAT. [HSA] = 2 μM, [CAT] = 2 μM and [TGC] = 0 to 1 x 10^{-5} M. X represents buffer only.

1.3. Fluorescence emission spectra of HSA-TGC-DIO system

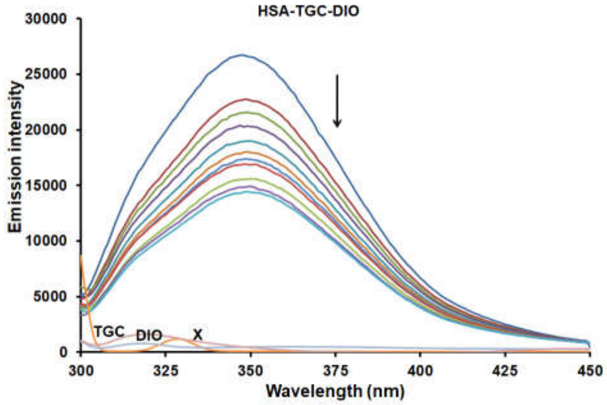

Figure S5. Fluorescence emission spectra of HSA-TGC (T = 298 K, pH = 7.4) in the presence of DIO. [HSA] = 2 μM, [DIO] = 2 μM and [TGC] = 0 to 1 x 10^{-5} M. X represents buffer only.

1.4. Stern-Volmer plots of the fluorescence quenching of HSA-TGC system by ALK and FLAVs

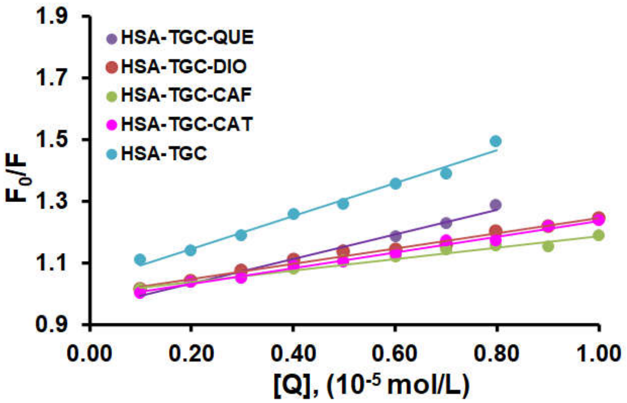

Figure S6. Stern-Volmer plots of the of the fluorescence quenching of HSA by TGC in the presence of CAF/FLAVs (QUE, CAT and DIO) at 298 K

1.5. Synchronous fluorescence emission spectra of HSA-TGC-CAF system

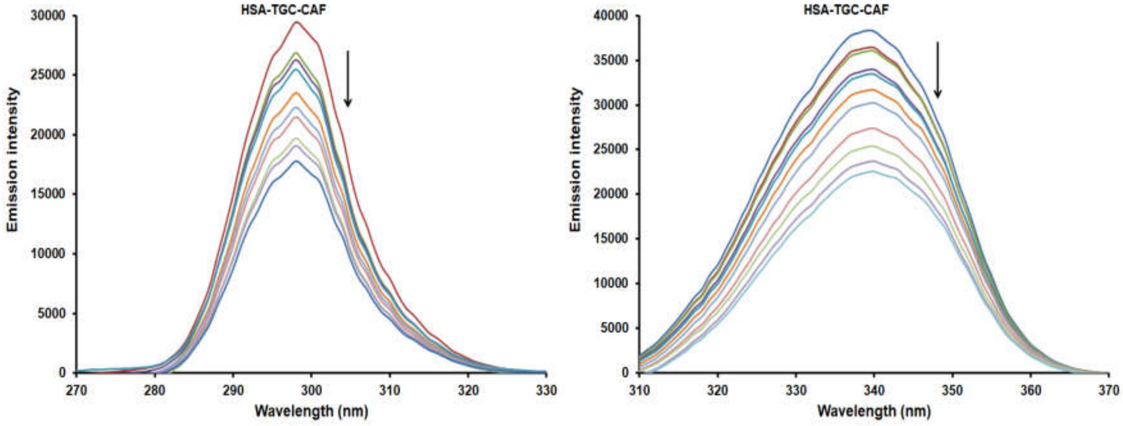

(a) (b)

Figure S7. The effect of CAF on the synchronous fluorescence emission spectra of HSA-TGC system (T = 298 K, pH = 7.4): a) $\Delta\lambda$=15 nm and b) $\Delta\lambda$=60 nm. [HSA] = 2 μM, [CAF] = 2 μM and [TGC] = 0 to 1 x 10^{-5} M.

1.6. Synchronous fluorescence emission spectra of HSA-TGC-CAT system

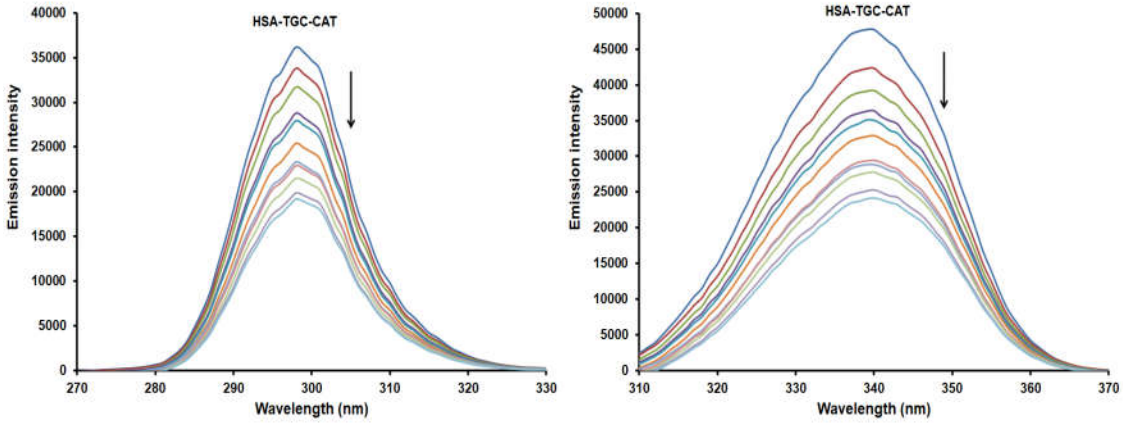

(a) (b)

Figure S8. The effect of CAT on the synchronous fluorescence emission spectra of HSA-TGC system (T = 298 K, pH = 7.4): a) $\Delta\lambda$=15 nm and b) $\Delta\lambda$=60 nm. [HSA] = 2 μM, [CAT] = 2 μM and [TGC] = 0 to 1 x 10^{-5} M.

1.7. Synchronous fluorescence emission spectra of HSA-TGC-DIO system

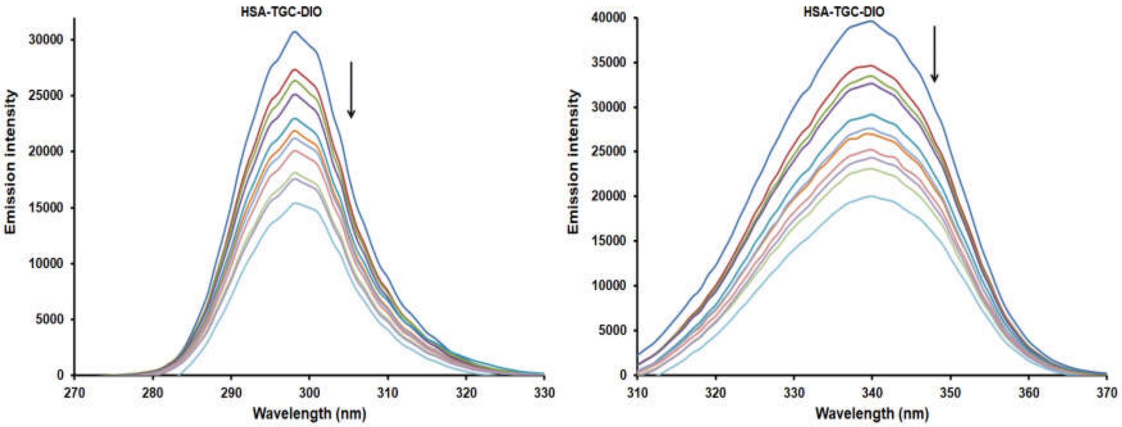

(a) (b)

Figure S9. The effect of DIO on the synchronous fluorescence emission spectra of HSA-TGC system (T = 298 K, pH = 7.4): a) $\Delta\lambda$=15 nm and b) $\Delta\lambda$=60 nm. [HSA] = 2 μM, [DIO] = 2 μM and [TGC] = 0 to 1 x 10^{-5} M.

1.8. FT–IR spectra of HSA in presence and absence of TGC and CAT

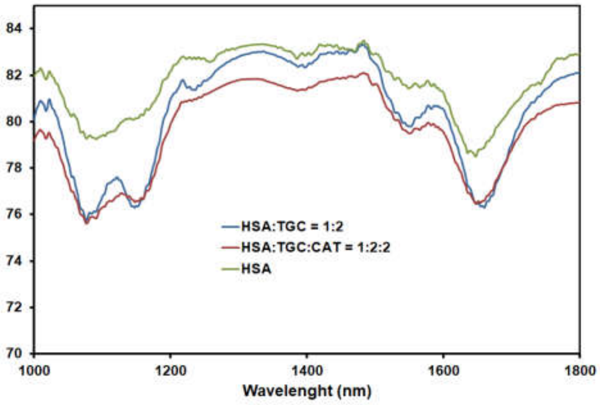

Figure S10. FT-IR spectra (wavenumber = 1000–1800 cm^{-1}) for HSA (2 μM) in presence and absence of TGC and CAT (4 μM)
